# Formation of Zwitterionic and Self-Healable Hydrogels via Amino-yne Click Chemistry for Development of Cellular Scaffold and Tumor Spheroid Phantom for MRI

**DOI:** 10.1021/acsami.4c06917

**Published:** 2024-07-08

**Authors:** Cao Tuong Vi Nguyen, Steven Kwok Keung Chow, Hoang Nam Nguyen, Tesi Liu, Angela Walls, Stephanie Withey, Patrick Liebig, Marco Mueller, Benjamin Thierry, Chih-Tsung Yang, Chun-Jen Huang

**Affiliations:** † Department of Chemical & Materials Engineering, National Central University, Jhong-Li, Taoyuan 320, Taiwan; ‡ R&D Center for Membrane Technology, Chung Yuan Christian University, 200 Chung Pei Road, Chung-Li City 32023, Taiwan; § Clinical Research and Imaging Centre, South Australian Health and Medical Research Institute, Adelaide 5001, Australia; ∥ Future Industries Institute, University of South Australia, Mawson Lakes Campus, Adelaide, SA 5095, Australia; ⊥ Siemens Healthcare Pty Ltd., Adelaide 3123, Australia; # Siemens Healthcare GmbH, Erlangen 91052, Germany; ∇ Advanced Clinical Imaging Technology, Siemens Healthineers International AG, Lausanne 1000, Switzerland

**Keywords:** amino-yne click reaction, self-healing, degradation, cell encapsulation, chemical exchange saturation transfer, magnetic resonance imaging

## Abstract

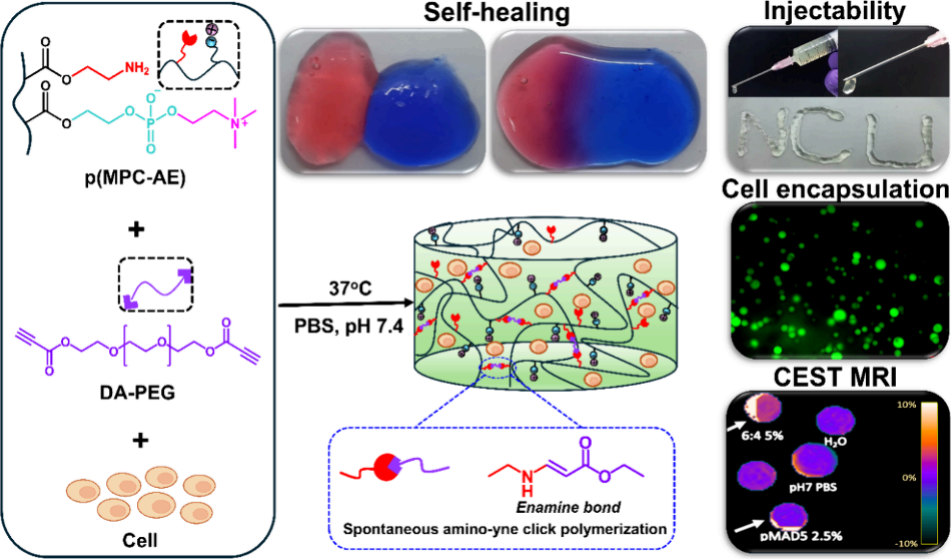

In situ-forming biocompatible hydrogels have great potential in various medical applications. Here, we introduce a pH-responsive, self-healable, and biocompatible hydrogel for cell scaffolds and the development of a tumor spheroid phantom for magnetic resonance imaging. The hydrogel (pMAD) was synthesized via amino-yne click chemistry between poly(2-methacryloyloxyethyl phosphorylcholine-*co*-2-aminoethylmethacrylamide) and dialkyne polyethylene glycol. Rheology analysis, compressive mechanical testing, and gravimetric analysis were employed to investigate the gelation time, mechanical properties, equilibrium swelling, and degradability of pMAD hydrogels. The reversible enamine and imine bond mechanisms leading to the sol-to-gel transition in acidic conditions (pH ≤ 5) were observed. The pMAD hydrogel demonstrated potential as a cellular scaffold, exhibiting high viability and NIH-3T3 fibroblast cell encapsulation under mild conditions (37 °C, pH 7.4). Additionally, the pMAD hydrogel also demonstrated the capability for in vitro magnetic resonance imaging of glioblastoma tumor spheroids based on the chemical exchange saturation transfer effect. Given its advantages, the pMAD hydrogel emerges as a promising material for diverse biomedical applications, including cell carriers, bioimaging, and therapeutic agent delivery.

## Introduction

Hydrogel-based materials, characterized by their high water content, transparency, biocompatibility, and resemblance to the extracellular matrix,^1−4^ have gained prominence in various biological applications such as drug delivery, 3D scaffolds, injectable tissue engineering, surgical glues, and tissue sealants.^5−10^ For example, hydrogel-based delivery systems have been designed to encapsulate and deliver bioactive factors such as macromolecules, drugs, and cells, and enhance their distribution to specific targets.^11^ Cell encapsulation within hydrogels is especially promising, providing environments that support normal cell function and protect them from external factors.^12^ However, the gelation process, material compositions, and mechanical properties of hydrogels affect the loading ability as well as cell viability, which tend to limit the practical application of hydrogels (Table S1).^13^

Many hydrogel-based materials have been developed for drug delivery to brain tumors. However, there is an unmet need for noninvasive monitoring of the multiconstituents of the hydrogel after implantation. Chemical exchange saturation transfer (CEST) imaging is an advanced magnetic resonance imaging (MRI) method that probes for chemical compounds and metabolites related to the body’s physiological function and pathological conditions, that can support cancer diagnosis and treatment.^14−16^ In CEST MRI, the magnetization of the water-exchangeable amide proton pool of proteins and peptides in tissue is first saturated using radio frequency (RF) saturation labeling. Next, the chemical exchange of the saturated amide protons with bulk water protons transfers this saturation to the bulk water pool, resulting in a reduction of the bulk water MR signal proportional to the targeted protein and peptide concentration. This process is repeated multiple times to enhance the attenuation effect before the bulk water signal is read out using an anatomical MRI sequence. By varying the frequency of the applied RF labeling pulse, a Z-spectrum of the bulk water signal is sampled for multiple RF frequencies around the bulk water absorption peak, which is then analyzed to create a quantitative map of the relative concentration of specific proteins and peptides in tissue. CEST MRI relies on the chemical properties of metabolites with various functional groups, such as amine, amide, and hydroxyl.^17^ Amide proton transfer (APT)-weighted CEST of hydrogel-based materials is widely explored in a preclinical setting for brain cancer imaging and treatment response assessment.^18,19^

In situ-formed hydrogels, transforming between sol–gel states under mild conditions, bears significant potential as in vivo cell scaffolds. In situ hydrogel is usually accompanied by self-healing properties that extend the longevity of implanted biomaterials.^20−22^ Self-healing materials enable them to repair structural damages and restore bulk properties in response to environmental stress. Hydrogels with both in situ-forming and self-healing properties can be prepared using chemical cross-linkers, enzymes for biological cross-linkers, physical interactions, and supramolecular chemistry.^23−26^ However, physically cross-linked hydrogels have poor mechanical properties and inflexible behaviors toward variables such as gelation time, chemical functionalization, and degradation.^27^ In addition, many hydrogels formed by chemical reactions face limitations in biomedical applications because of residual byproducts from the hydrogel formation, such as residual photoinitiators, monomer molecules, catalysts, and solvent that cause toxicity to cells.^28^ Therefore, there is a need to develop novel, safe cross-linkers that ensure the mechanical stability and biocompatibility of the hydrogel for biomedical applications.

To address biocompatibility issues, a novel cross-linking system using amino-yne click polymerization has been developed. The method leads to more efficient and environmentally friendly cross-linking than other click chemistry methods (comparative analysis of different click chemistries is shown in Table S2). For example, the Huisgen 1,3-dipolar cycloaddition reaction between an azide and a terminal alkyne is the most popular click reaction, which usually requires high temperatures and Cu (I) as a catalyst. Commonly used solvents for this reaction are polar aprotic solvents such as tetrahydrofuran (THF), dimethyl sulfoxide (DMSO), acetonitrile, and dichloromethane (DCM).^29^ Nevertheless, the amino-yne system operates at ambient temperature without the need for catalysts or stimuli, resulting in nontoxic hydrogels.^30^ The spontaneous amino-yne click reaction has been demonstrated for its reliability and effectiveness in mild conditions.^31^ Notably, it offers the advantage of occurring without the need for organic solvents, making it suitable for in vivo applications.^32^ Synthetic polymers offer precise control of the mechanical and desirable characteristics of hydrogels_._^33,34^ Zwitterionic polymers, with oppositely charged groups in the same moiety, exhibit high hydrophilic and biocompatible behaviors, along with excellent antifouling performance.^35−39^ Particularly, zwitterionic polymers afford promising self-healing behavior through electrostatic forces between anionic and cationic groups, leading to a long lifespan and enhancing the durability of the material.^40,41^

**Scheme 1:**
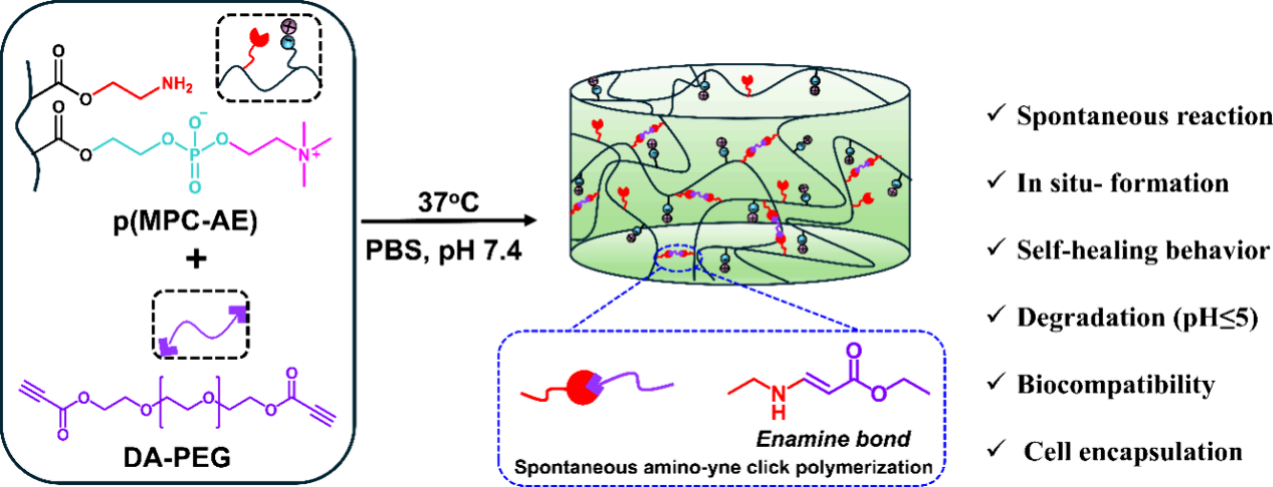
Schematic Illustration of In Situ-Forming Hydrogel pMAD via Spontaneous Amino-yne Click Reaction under a Physiological Condition at 37 °C and pH 7.4

This work presents a novel hydrogel prepared via the amino-yne reaction, demonstrating good mechanical, self-healing, and biological properties (Scheme 1). Random copolymers of poly(2-methacryloyloxyethyl phosphorylcholine-*co*-2-aminoethylmethacrylamide) (pMA) were synthesized, forming in situ, biocompatible, and degradable hydrogels (pMAD) with the dialkyne polyethylene glycol (DA-PEG) cross-linker. These hydrogels are promising candidates for cell encapsulation and could also be assessed post implantation using CEST MRI. Characterization of the pMA copolymers and DA-PEG cross-linkers was performed using nuclear magnetic resonance (NMR), attenuated total reflectance Fourier-transform infrared (ATR-FTIR) spectroscopies, and gel permeation chromatography (GPC). The gelation of the pMAD hydrogel was assessed by the inverted vial method and rheology analysis. Physical and biological properties, including compressive mechanical properties, equilibrium water content, degradability, cell cytotoxicity, and cell encapsulation with NIH-3T3 fibroblasts, were characterized. The U87 cell line was used to generate GBM spheroids to establish the feasibility of the pMAD hydrogel as a promising candidate for in vivo CEST MRI.^42^ The in situ-forming pMAD hydrogel demonstrates capabilities for various medical applications.

## Materials and Methods

### Materials

2-Methacryloyloxyethyl phosphorylcholine (MPC, 97%) and 2-aminoethyl methacrylate hydrochloride were purchased from Sigma-Aldrich. Poly (ethylene glycol) (PEG, *M*_w_ = 2 kDa), propiolic acid, p-toluenesulfonic acid monohydrate (p-TSA), toluene, diethyl ether, 2-propanol, azobisiobutyronitrile (AIBN), and DMSO were supplied by ACROS Organics. Fetal bovine serum (FBS), Dulbecco’s Modified Eagle Medium (DMEM), and the LIVE/DEAD Viability/Cytotoxicity Kit were obtained from Thermo Scientific.

### Synthesis of Dialkyne Polyethylene Glycol Cross-Linker

The cross-linker dialkyne polyethylene glycol was synthesized according to previous literature published by Huang et al.^43^ The mixture of PEG (10.0 g, 5 mmol), propiolic acid (3.5 g, 50 mmol), and p-TSA (0.57 g, 3 mmol) were dissolved in dry toluene (150 mL). After being stirred and refluxed for 48 h, the mixture was concentrated under a vacuum. The solution was precipitated with diethyl ether and recrystallized in isopropanol. The resulting powder was collected and dried in vacuum. The DA-PEG cross-linker was obtained as a light-yellow powder, yielding 79% (Scheme S1).

### Synthesis of (2-Methacryloyloxyethyl Phosphorylcholine)-*co*-(2-aminoethyl Methacrylate Hydrochloride) Polymer (pMA)

Free radical polymerization was chosen to prepare a copolymer that combined MPC and AE monomers with molar ratios of 5:5, 6:4, and 7:3 (Table 1). AIBN was used as the initiator. Briefly, MPC (0.885 g, 3.0 mmol) and AE (0.5 g, 3 mmol) were dissolved in 18 mL of DI water in a round bottle. Then, AIBN (0.005 g, 0.03 mmol) was dissolved in 2 mL of DMSO and added to the above mixture. The final mixture was bubbled with nitrogen for 30 min and then heated at 70 °C for 24 h. To remove the unreacted monomer, the polymer solution was dialyzed using a cellulose membrane (MWCO 6–8 kDa) and freeze-dried. Then, the copolymers were analyzed by nuclear magnetic resonance spectroscopy (^1^H NMR, 600 MHz, in D_2_O) (Scheme S1).

**Table 1:** Molecular Compositions of pMA Copolymers

copolymer	MPC:AE ratio
pMA5	5:5
pMA6	6:4
pMA7	7:3

### Formation of Hydrogel

The pMAD hydrogels were prepared by spontaneous amino-yne click polymerization at 37 °C. The different concentrations of pMA solution (0.5–4.5 wt %) were initially prepared by dissolving copolymers in phosphate-buffer saline (PBS). The DA-PEG cross-linker was added to a glass vial containing 1 mL of a copolymer solution. Then, the precursor solution was mixed homogeneously and incubated at 37 °C for 24 h. Gelation time and concentration were measured by the inverted vial method at various time points.^44^ The compositions of the hydrogel synthesized are shown in Table 2.

**Table 2:** Molecular Compositions of pMAD Hydrogels

no	sample	copolymer	pMA concentration (wt %)	cross-linker concentration (wt %)
1	pMAD542	pMA5	4	2
2	pMAD550		5	0.5
3	pMAD551			1
4	pMAD552			2
5	pMAD562		6	2
6	pMAD572		7	2
7	pMAD650	pMA6	5	0.5
8	pMAD651			1
9	pMAD652			2
10	pMAD662		6	2
11	pMAD662		7	2
12	pMAD750	pMA7	5	0.5
13	pMAD751			1
14	pMAD752			2
15	pMAD762		6	2
16	pMAD772		7	2

### Gel Permeation Chromatography (GPC)

The molecular weight (*M*_w_) and polydispersity index (PDI = *M*_w_/*M*_*n*_) were determined using aqueous GPC (Viscotek GPC Max Module, Houston, USA). The system utilized a Viscotek refractive index detector with a Viscogel G6000 PW XL column, operating at a flow rate of 1.0 mL min^–1^ and a temperature of 40 °C. The eluent was a 0.1 M NaNO_3_ aqueous solution. Calibration was performed using poly (ethylene oxide) standards, with the molecular weight ranging from 3450 to 217,000 Da (7 points). Prior to the experiments, all samples (1–0.2 wt %) were filtered through a 0.22 μm PPTFE filter.

### Rheological Test

A stress-controlled rheometer was used to conduct the rheological test, measuring the formation of pMAD hydrogel. Since the gelation mechanism is consistent across all samples, the fastest gelation, pMAD552, was chosen for rheological testing to confirm the success of gelation. In detail, pMAD552 hydrogel with 5 wt % of pMA5 copolymer and 2 wt % of DA-PEG cross-linker were prepared and stored at 4 °C. The hydrogel precursor solution was loaded between parallel plates with a diameter of 40 mm and a gap of 0.5 mm, and the temperature was maintained at 37 °C during the measurement. The storage modulus (*G*′) and loss modulus (*G*″) of hydrogels were characterized as a function of frequency at 1% strain and 1 Hz of frequency.

### Compressive Mechanical Test

Hydrogel samples were prepared with different mole ratios between MPC and AE at 37 °C for 24 h. The precursor solution was poured into cylinder molds (7 mm in diameter and 14 mm in height). After removing the molds, the hydrogels were placed on the compression plate. The mechanical properties were measured by a mechanical tester (Instron 4467, Instron Co., USA) at 5 mm/min with a 10 N load cell. The modulus was calculated from 10 to 20% strain of the stress–strain curve.

### Swelling Capacity

The swelling capacity of hydrogel was measured by gravimetric analysis. First, pMAD hydrogels were lyophilized until completely dry. Then, lyophilized samples were immersed in PBS (pH 7.4) for 3 days at 37 °C. The swelling ratio of the samples was obtained from the below equation:
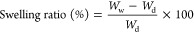
1where *W*_w_ is the mass in the swollen state and *W*_d_ is the mass in the dried state of the hydrogel. The experiment was repeated three times.

### Degradability Test

The pMAD hydrogels with 0.5, 1, and 2 wt % concentrations of DA-PEG cross-linker concentration were immersed in buffer at various pH values (2, 5, 7.4, and 8) at 37 °C. At the specific time, the weight of the remaining hydrogel was determined by gravimetric analysis.
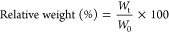
2where *W*_t_ is the degraded weight and *W*_0_ is the initial dry weight.

### Self-Healing Test

The morphology of hydrogels was characterized through observation.^45,46^ pMAD552 hydrogels were used to measure self-healing behavior. Without any external stimuli, we put two hydrogel pieces in contact with one another. One piece of hydrogel was dyed red and the other left blue to observe the interface diffusion phenomena. The degree of fusion reflects the size of contact between two cuts as well as the mobility of the cross-linked network.

### Cytotoxicity Test

The MTT assay was used to evaluate the cytotoxicity of pMAD hydrogels to NIH-3T3 fibroblasts. All samples were sterilized by soaking in a 70% ethanol solution for 30 min and washing three times with PBS. Sterilized pMAD hydrogels were immersed in Dulbecco’s Modified Eagle’s Media (DMEM) at 37 °C overnight to get extraction for the cytotoxicity test. NIH-3T3 fibroblasts were cultured in each well of a 96-well plate with a seeding concentration of 1 × 10^4^ cell/mL in DMEM containing 10% FBS at 37 °C for 24 h. Afterward, the cell medium was replaced with the 300 μL of hydrogel extraction solution and incubated at 37 °C for 1 day. After incubation, 30 μL (5 mg/mL in PBS) MTT solution was added to each well and incubated for another 3 h. Finally, the medium was removed, 300 μL of DMSO was added to each well, and the absorbance at OD_540nm_ was measured using the ELISA reader (Synergy 2, Bio Tek, USA). The stated value is the average obtained from three replicates and shown as a percentage compared to the control samples.

### Cell Encapsulation

Cell encapsulation of pMAD hydrogels was tested with NIH-3T3 mouse fibroblast at a seeding concentration of 2 × 10^4^ cells/mL in a serum-free DMEM at 37 °C. Initially, the copolymer pMA5 and DA-PEG cross-linker were dissolved in DMEM medium and mixed to prepare the pMAD552 precursor hydrogel. After 10 min of gelation, NHI-3T3 cells were added and suspended in a precursor solution, and they were then incubated in a 24-well plate at 37 °C for 24 h to encapsulate cells. Finally, one group of samples was added to serum-free DMEM media to provide a favorable environment for cell growth. The other group conducted the same as the first one, but the difference was using DMEM that contained 10% FBS, which supplied nutrients for cell growth. Both samples were cultured in a 24-well plate at 37 °C. The cell encapsulation efficiency was determined after 3, 5, and 7 days in the former circumstance. The number of cells and the proliferation rate were observed after 1, 3, and 5 days. The LIVE/DEAD Viability Kit was used to observe the growth of cells in the hydrogel under a fluorescent microscope (Nikon Eclipse, Ts2-FL, Japan). The cell numbers were estimated using ImageJ software.

### Phantom Preparation and MRI Experiments

The pilot MRI experiment aimed to investigate the capability of the pMAD hydrogel to demonstrate the CEST effect at pMA5 copolymer concentrations of 2.5–5 w/t% with an increase of 0.5 w/t%, using known reference samples such as phosphate-buffered saline solution (PBS, pH 7), raw egg white, cooked egg, and various concentrations (30, 65, and 100%) of egg white protein (Paleo Protein Powder, Protein Supplies Australia).^47,48^ Subsequently, a second MRI experiment was conducted to compare the effects of the pMA5, pMA6, and pMA7 copolymers of the pMAD hydrogel at concentrations ranging from 2.5 to 5.0 wt % with an increment of 0.5 wt %. Finally, a U87 tumor spheroid phantom was prepared by placing a U87 spheroid at the maximum diameter of approximately 1.5 mm with a cross-sectional area of 1.77 mm^2^ in solutions containing pMAD hydrogel with pMA5 copolymer at 4.0 and 5.0 wt %, respectively. Polydimethylsiloxane (PDMS) was dispensed on the bottom of the vials to bring the interface of the spheroids up to the middle of the vial to obtain better MRI images, and a PBS solution was used as the control.

A 3D gradient-echo research APT-weighted CEST MRI sequence was performed on a MAGNETOM Skyra 3T MR scanner (Siemens Healthcare; Erlangen, Germany) using a 64-channel head/neck coil. CEST maps of the phantom were acquired with flip angle = 7°, TR = 4.11 ms, TE = 2.08 ms, FOV = 220 × 178 mm, matrix size = 128 × 104 interpolated to 256 × 208, compressed sensing acceleration factor = 5, bandwidth = 700 Hz/pixel, and a total of 12 slices within a 5 mm slice thickness. The RF saturation pulse train consisted of 36 Gaussian-shaped RF pulses, *t*_pulse_ = 50 ms, *t*_delay_ = 5 ms, *T*_sat_ = 2.0 s, DC_sat_ = 91%, B1 = 2.02 μT, and relaxation time = 2400 ms. A total of 30 z-spectral points were sampled with the saturation offsets using a 0.5 ppm increment from −6 to +6 ppm around the bulk water absorption peak, with additional sampling at the expected amide proton absorption peak at ±3.5 ppm. The total acquisition time was 5 min and 40 s. A *B*_0_ map was acquired for postprocessing. Data were processed using the CEST-EVAL software (German Cancer Research Center, DKFZ, Heidelberg, Germany) written in Matlab (R2021a, The MathWorks, USA). The Z-spectrum data were corrected for B_0_ inhomogeneity and motion directly on the scanner. Regions of interest (ROIs) were manually drawn on APT-CEST maps on three continuous slices. Mean APT-WEIGHTED CEST values were calculated by averaging across the three slices.

### Statistical Analysis

The data were reported as means ± standard deviation (SD) or standard error of the mean (SEM). Student’s *t* test was utilized to determine the statistical analyses among different groups. The probabilities of *p* ≤ 0.05 were considered significant.

## Results and Discussion

### Synthesis of pMA and DA-PEG

**Figure 1:**
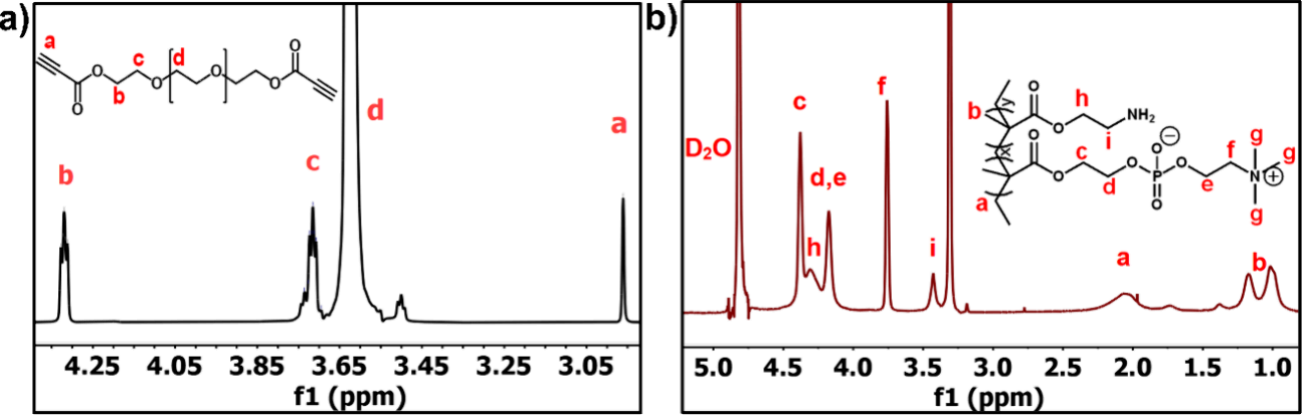
(a) ^1^H NMR spectra for DA-PEG and (b) ^1^H NMR spectrum of copolymer pMA5 in D_2_O.

The hydroxyl-terminated PEG was esterified with propiolic acid by Fischer esterification to produce DA-PEG with a yield of 80%. The structure of DA-PEG was confirmed by the ^1^H NMR spectrum (Figure 1a). The presence of the ethynyl proton (−C≡C−) and the methylene proton (−CH_2_−) next to the ester group was indicated by the new peaks appearing at 2.96 and 4.32 ppm, respectively. Moreover, the backbone PEG in the cross-linker is confirmed by the peak at 3.6 ppm (−CH_2_–CH_2_–O−). Figure S1 illustrates the PEG structure with the IR bands at 1465 and 1343 cm^–1^, which are assigned to the C–H bending. Additionally, the stretching signals for −OH and C–O–H were observed at 1280, 1236, and 1104 cm^–1^. Besides, new peaks appeared at 2112 and 1715 cm^–1^, presented for–C≡C– and −COO^–^, respectively. Therefore, the DA-PEG cross-linker was successfully synthesized by incorporating an alkyne group of propiolic acid into the polyethylene glycol structure. The results are in agreement with the previous work by Huang and co-workers.^43^

**Figure 2:**
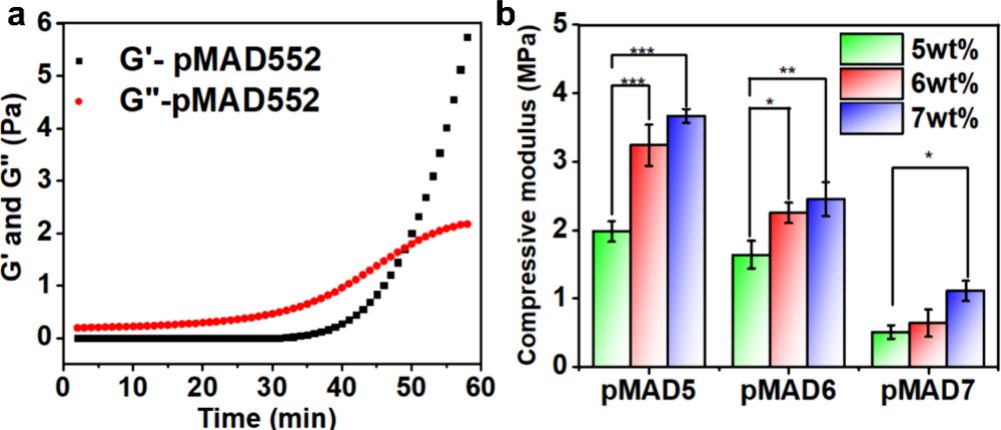
(a) *G*′ and *G*″ for the formation of hydrogel pMAD552 with the gelation time, measured data frequency of 1 Hz at 37 °C by rheological analysis with a strain of 1%. (b) Compressive modulus of the pMAD hydrogel with a constant 2 wt % DA-PEG cross-linker (*p < 0.05, **p < 0.01, and ***p < 0.001).

According to the FTIR spectrum in Figure S2, the peak in the pMA copolymer centered at around 1704 cm^–1^ is attributed to the −COO– of the ester group, while the peaks at 1228, 1061, and 956 cm^–1^ are assigned to −O–P–O–,–P–O–C–, and–N^+^(CH_3_)_3_ functional groups, respectively, demonstrating the presence of MPC. The small absorptions at 1667 and 789 cm^–1^ indicate the presence of–NH_2_ groups from the poly(AEMA) segments.^49^ The ^1^H NMR spectra of pMA in D_2_O are shown in Figure 2a. ^1^H NMR (CDCl_3_, 600), δ (ppm) = 1.18 ppm: (b, CH_3_, 3H), 2.06 ppm (a, CH_2_, 2H), 3.31 ppm (g, N(CH_3_)_3_,3H), 3.43 ppm (i, CH_2_NH_2_,2H), 3.76 ppm (f, CH_2_N(CH_3_)_3_,2H), 4.18 ppm (d,e, OCH_2_,2H), 4.32 ppm (h, OCH_2_CH_2_NH_2_,2H), 4.38 (c, OCH_2_CH_2_O, 2H). Copolymers pMA were synthesized with three feed ratios, including 5:5, 6:4, and 7:3, which were named pMA5, pMA6, and pMA7, respectively. From the ^1^H NMR spectrum, the actual ratio and conversion rate were calculated from the ratio of the integral peak areas of i from AE and e from MPC (Figure S3). Table 3 lists the feed composition, conversion rate, and molecular weights of the pMA copolymers, as well as their specific degree of polymerization.

**Table 3:** Characterization of Copolymer pMA with Different Ratios of MPC:AEMA

copolymers	conversion	feed ratio	actual ratio^a^	*M*_w_^a^	*M*_*n*_^b^	*M*_w_/*M*_*n*_^b^
pMA5	95.4%	5:5	5:3.6	32166	14131	2.27
pMA6	97.7%	6:4	6:2.9	42518	20759	2.04
pMA7	87.6%	7:3	7:2.1	76674	54189	1.41

a Determined by 600 MHz NMR.

b Determined by GPC, poly(ethylene oxide) as the standard.

### Formation of Hydrogel pMAD

The rheological characteristics of the pMAD552 hydrogel were determined by a dynamic viscoelastic method with a frequency of 1 Hz at 37 °C. In Figure 2a, the loss modulus (*G*″) initially showed a higher value than the storage modulus (*G*′), indicating that the hydrogel had not yet formed. After 50 min, the curve of *G*′ increased to reach the crossover point with *G*″, corresponding to the gelation point of the pMAD552 hydrogel. Consequently, the cross-linking between copolymer pMA5 and cross-linker DA-PEG proceeded within 50 min by the formation of enamine bonds under mild conditions. Previous work with amino-yne click polymerization was conducted in hazardous organic solvents (i.e., THF and DCM).^32^ Herein, the rheological results of pMAD hydrogel indicate that under physiological settings (37 °C, PBS buffer pH 7.4), the formation of enamine linkages can occur between the terminal alkyne groups of DA-PEG and the amino groups of the pMA via amino-yne click polymerization without the need for a catalyst, initiator, or additional energy.

Accordingly, the effects of the concentration of the pMA copolymer and the cross-linker DA-PEG on gelation were determined using the inverted vial method. The precursors for the pMAD hydrogel were incubated at 37 °C, and the gelation time was recorded over 24 h. After inverting the vial, the sample was considered a hydrogel if there was no flow after 5 min. This test is conducted by visual observation and tactile sensation to determine the sol-to-gel transition.^41^

As shown in Figure S4, the gelation time can be tuned from about 2 h to 24 h by adjusting copolymer and cross-linker concentrations. Generally, pMAD hydrogels prepared with the pMA5 copolymer showed the fastest gelation, while using the pMA7 copolymer required more time for forming the hydrogel because of the low molar percent of the amino moieties (AE) in the copolymer. The analysis of the gelation diagram in Figure S4 revealed that the minimum concentration for forming the hydrogel was 2 wt % for the pMA5 copolymer and 0.5 wt % for the DA-PEG cross-linker. With the same concentration of DA-PEG, the gelation point was obtained with 5 wt % pMA6 copolymers, which showed a higher needed concentration than pMA5, and there was no gelation of pMA7 copolymer with 0.5 wt % cross-linker. Therefore, pMAD hydrogel formation is related to the amount of the AE moieties in the pMA copolymer.

The gelation not only depends on the number of −NH_2_ moieties present in the copolymer but is also influenced by adjusting the ratio between copolymer and cross-linker. As shown in Figure S4, the gelation time decreased with increases in the copolymer and cross-linker concentrations, owing to the increases in the density of end groups in the final network. The ability to adjust the spatiotemporal control of gelation time via regulating hydrogel components, including copolymer and cross-linker concentrations as well as the molar ratio between MPC and AE moieties, is highly appealing for injectable hydrogels and other biomaterial applications.

### Mechanical Test

The mechanical properties of the pMAD hydrogel were determined by tuning the MPC:AE molar ratio, and copolymer and cross-linker concentrations. First, we depict the increased tendency of the compressive modulus by using a series of ascending copolymer concentrations, including 5, 6, and 7 wt %. It is worth noting that the higher the concentration of copolymer used, the better the mechanical properties of the hydrogel. Specifically, the compressive modulus of pMAD572 was 3.69 ± 0.03 MPa, and that of pMAD552 decreased obviously to 1.98 ± 0.15 MPa. Moreover, the modulus of the pMAD hydrogel using the pMA5 copolymer was significantly higher than that of pMA6 and pMA7 (Figure 2b). Denser hydrogel networks were formed as a result of regulating the number of AE units in the copolymer structure, leading to an increased probability of cross-linking density.

To further evaluate the effect of the DA-PEG concentration on the mechanical properties, pMAD hydrogels were prepared at a fixed polymer concentration of 5 wt %, while the concentration of DA-PEG cross-linker was varied from 0.5, 1, to 2 wt %. As shown in Figure S5d–f, the compressive strength of the pMAD hydrogel increased with the increase in the cross-linker concentration. Consequently, the mechanical properties of pMAD hydrogels depend on the predominant number of alkyne groups in the DA-PEG cross-linker. According to the result of the compressive test and the gelation of the hydrogel shown in Figures S5 and S6, respectively, the pMAD hydrogel, which was prepared from a 0.5 wt % DA-PEG cross-linker, has a highly swollen structure (pMAD550, pMAD650, and pMAD750). Thus, the mechanical properties of the pMAD hydrogel can be optimized by adjusting the concentration of components in the hydrogel.

### Swelling and Degradation of Hydrogels

**Figure 3:**
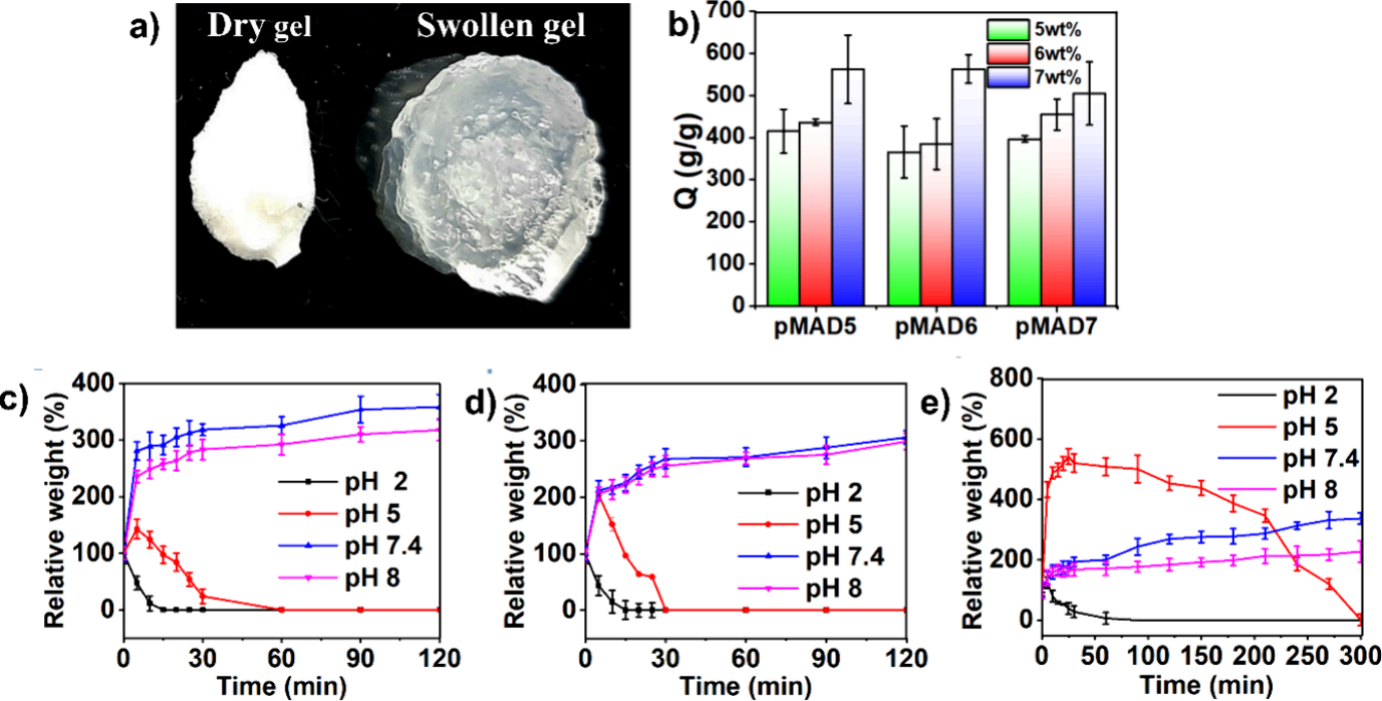
(a) Photo of dry and swollen pMAD hydrogels and (b) swelling ratio Q of the pMAD hydrogel incubated in PBS at 37 °C. Degradable behavior of the pMAD hydrogel with different molar ratios of cross-linkers, (c) pMAD550, (d) pMAD551, and (e) pMAD552.

The swelling ratio was calculated by swelling a hydrogel at different copolymer concentrations in a PBS solution until it reached an equilibrium state (Figure 3a). The results presented in Figure 3b indicate that the swelling ratio of all pMAD hydrogels reached over 300% after 24 h, with the swelling ratios of the pMAD hydrogels increasing with increased copolymer concentrations. This can be attributed to the hydrophilicity of MPC and AE units in the hydrogel structure, which facilitates their binding to more water molecules. Additionally, the swelling ratios of the hydrogels are attributed to the capabilities of gels to adsorb water molecules via hydration, and absorb them via large water retention volume developed by expansion of polymer chains in water. Herein, no obvious difference in the swelling ratios of the pMAD hydrogels using pMA5, pMA6, and pMA7 copolymers could be due to offsetting effects among the hydrophilicity of the hydrogel compositions, polymer chain flexibility, pore sizes, and cross-linker spatial distribution.^50,51^

To assess the pH-responsive behavior of pMAD hydrogels, pH values of 2, 5, 7.4, and 8 were used to estimate the degradation rate of the hydrogel. As shown in Figure 3c–e, pMAD hydrogels were completely degraded after 15 min at pH 2 for all cross-linker concentrations and after 30 min at pH 5 at the lower cross-linker concentrations of 0.5 and 1 wt %. The degradation of the pMAD hydrogels in acidic conditions can be explained by the hydrolysis of enamine bonds in the hydrogel network (Scheme S2). Interestingly, pMAD552 hydrogel reached equilibrium swelling at the beginning of incubation and gradually degraded until 5 h later to achieve complete degradation at pH 5. The swelling behavior of pMAD552 was attributed to the formation of enamine bonds in the hydrogel network. In general, primary amines can react with alkynes through the hydroamination of alkynes in an amino-yne click reaction to form enamine bonds. Moreover, the enamine linkage is readily hydrolyzed back to aldehydes and a primary amine by breaking the C–N bonds under an acidic condition at a pH value below 6.8.^52−54^ The hydrogel with an amino group backbone was protonated and positively charged in acidic buffer at pH 5, and the swelling behavior of the hydrogel was contributed by electrostatic repulsion between positively charged −NH^3+^ groups. The pMAD hydrogel is designed to degrade under acidic conditions, making it effective for delivering stem cells and therapeutic agents (e.g., CAR-T cells and doxorubicin) to tumor sites with mildly acidic microenvironments (pH 6.5–6.9). This acidity results from elevated glucose metabolism, ion H+ production, and excretion. Although the degradation rate in the tumor environment is slower compared to pH ≤ 5, it enables the controlled and sustained release of cells over an extended period.^55^ This property is particularly advantageous for chronic conditions requiring prolonged treatment, as it reduces the need for frequent administration or replacement and provides sufficient time for cells to adapt and integrate into the surrounding tissue.^56^

### Self-Healing Test

**Figure 4:**
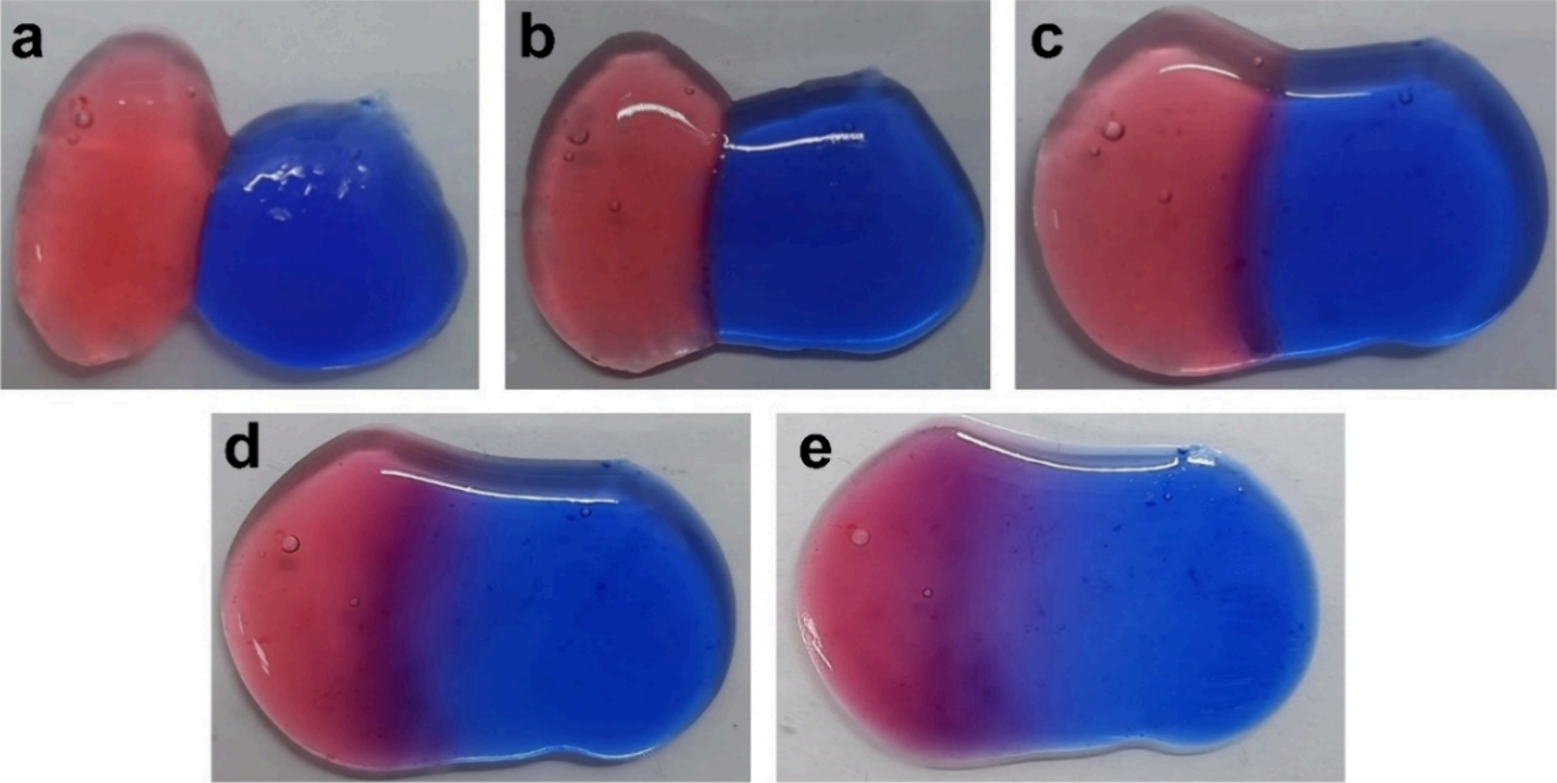
Optical assessment of the self-healing process of pMAD552 in (a) 0, (b) 15, (c) 30, (d) 60, and (e) 120 min.

**Scheme 2:**
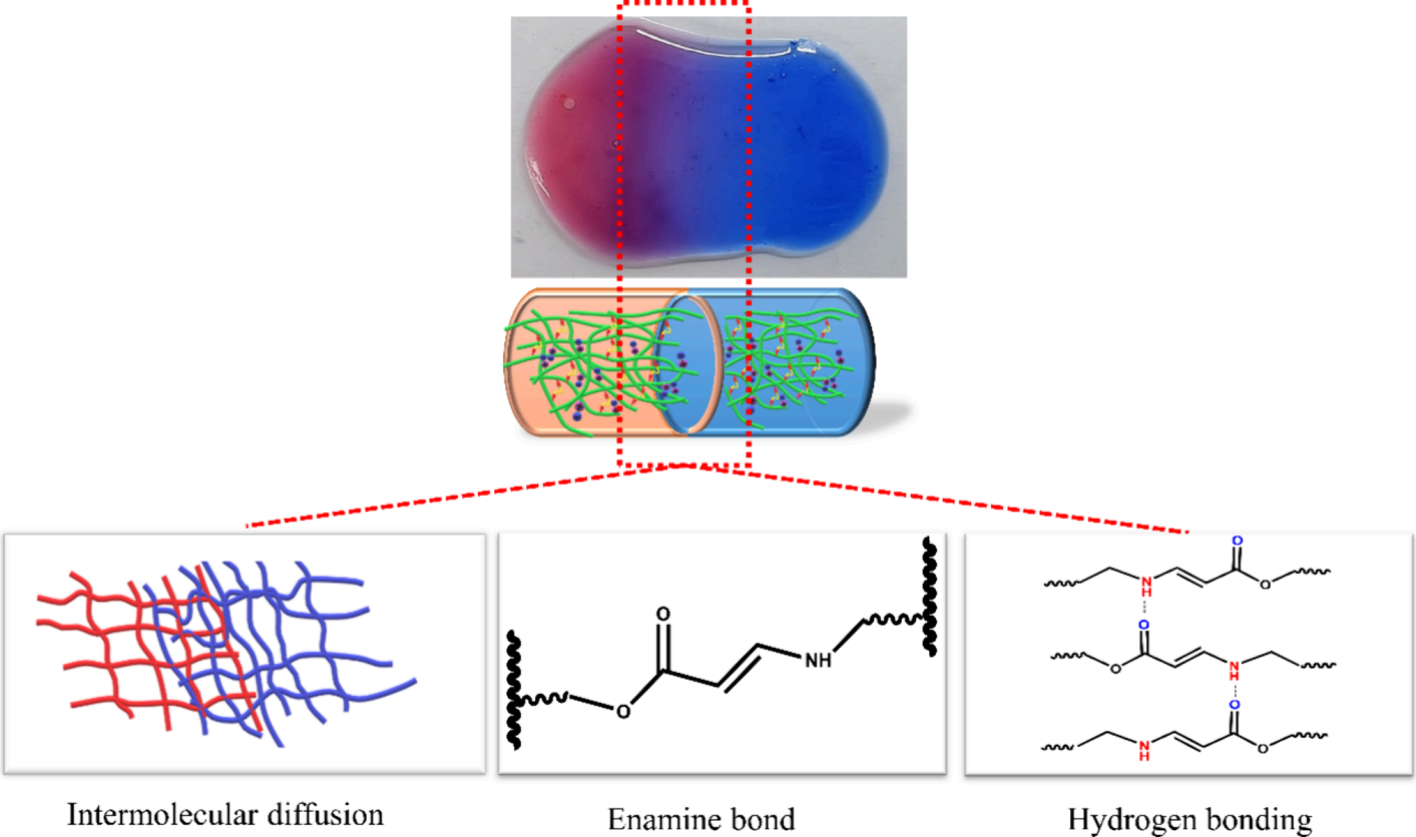
Self-Healing Mechanism of the Hydrogel under a Physiological Condition at pH 7.4

Self-healing materials are a type of smart material that has garnered significant interest recently, capable of restoring the structure after damage, thereby enhancing the overall performance and extending the lifetime of the materials. As shown in Figure 4, two differently colored pieces of the pMAD552 hydrogel were brought into contact. Without any external intervention and at room temperature, the hydrogel completely fused together after a few minutes. It can be observed that the border between the two pieces of hydrogels gradually blurred after 30 min of contact. The self-healing properties of the pMAD hydrogel are governed by the “dangling chain”, wherein the copolymer or cross-linker repairs the gel through both physical and chemical interactions. The diffusion of dyes across the interface demonstrated the healing behavior of the pMAD552 hydrogel. Initially, the hydrogel network recovers as a result of the polymer chains on the hydrogel surface continuing to move and cross over together.^57^ Subsequently, the self-healing performance of the pMAD552 hydrogel was achieved by forming dynamic enamine bonds and multiple hydrogen bonds between their interactions under physical conditions (7.4) (Scheme 2).^58^

### Cytotoxicity and Encapsulation Test

**Figure 5:**
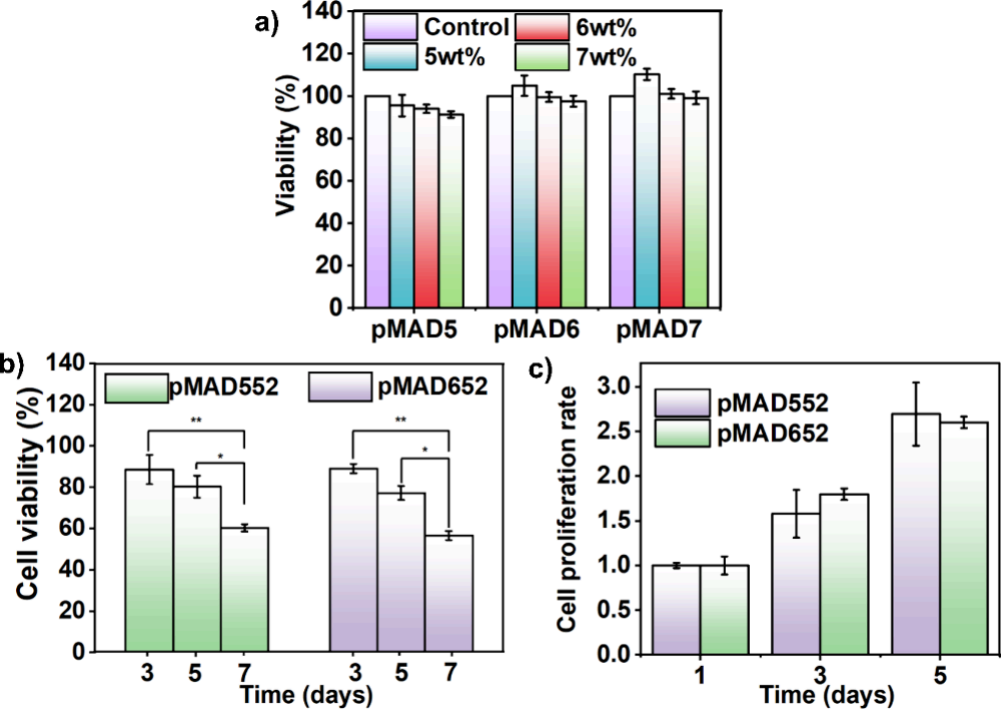
(a) Cell viability of NIH-3T3 fibroblasts in the pMAD hydrogel, (b) cell viability of NIH-3T3 fibroblasts in the pMAD hydrogel during 3,5, and 7 days in DMEM, and (c) cell proliferation of NIH-3T3 fibroblasts encapsulated in pMAD hydrogels at 1,3, and 5 days in DMEM (10% FBS) (* *p* ≤ 0.05, ** *p* ≤ 0.01).

The biocompatibility of the pMAD hydrogels was determined through an extraction cytotoxicity test using NIH-3T3 fibroblasts for 24 h. As presented in Figure 5a, all tested pMAD hydrogel samples with varying copolymer concentrations showed no toxicity, with viability values exceeding 80%. The excellent biocompatibility of pMAD is attributed to its nontoxic constituents. Notably, the negatively charged phosphate and positively charged choline groups of MPC resemble the polar groups of phospholipids in the cell membrane. Additionally, the polyethylene glycol structure of DA-PEG, commonly used in medical products, further supports the biocompatibility of pMAD hydrogel, making it suitable for biomedical applications.

To further investigate the potential for 3D cell encapsulation with the pMAD hydrogel, NIH-3T3 fibroblasts were suspended in pMAD hydrogel precursors, prepared by mixing 2 wt % DA-PEG cross-linker with 5 wt % pMA copolymers (pMA5, pMA6). Cell viability was evaluated using a LIVE/DEAD assay in serum-free DMEM solutions. As shown in Figure 5b, after 3 days of culture, nearly 90% of the cells remained alive in the pMAD552 and pMAD652 hydrogels, indicating that the amino-yne click reaction in the formation of the hydrogel had negligible effects on cells. The viability of NIH-3T3 cells in pMAD552 and pMAD652 hydrogels remained over 80% for up to 5 days, with no significant difference observed between the two hydrogels. Moreover, the decrease in cell count after 7 days of culture might be attributed to enzyme release from cells and hydrolytic degradation, resulting in partial degradation of the pMAD hydrogel. Nevertheless, the hydrogel successfully encapsulated and retained cells for up to 7 days.

For cell encapsulation in DMEM containing 10% FBS, cells were cultured in the hydrogel for 5 days, and fluorescent images were captured to track cell survival rate and proliferation in 3D hydrogels. The increasing cell population density in hydrogels demonstrated exceptionally high vitality throughout the growth process, confirming the biocompatibility of the pMAD hydrogels. Based on Figure 5c, the cell number increased considerably after 5 days of culture, with a substantial difference observed between days 1, 3, and 5. Consequently, FBS diffusion into the pMAD network stimulated the proliferation of NIH-3T3 cells. No significant difference in cell proliferation was observed between pMAD552 and pMAD652, indicating the marginal effect of the polymer compositions on cell culture (Figures S7 and S8).

### MRI Results

**Figure 6:**
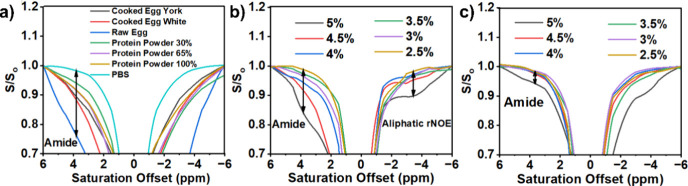
(a) Z-spectrum of PBS solution, 30, 65, and 100% egg white protein solution, raw egg and white solution, and cooked egg white and egg yolk. The Z-spectrum of the pMAD hydrogel with corresponding copolymers (b) pMAD5 and (c) pMAD6 concentration on 2.5 wt %, 3.0 wt %, 3.5 wt %, 4.0 wt %, 4.5 wt %, and 5.0 wt %. The amide 3.5 ppm peak and aliphatic rNOE are indicated. NOE: Nuclear Overhauser Effect.

Figure 6a illustrates the Z-spectrum plot derived from the MRI experiments conducted on the pMAD hydrogel using the pMAD5 copolymer and known reference samples. The results reveal a consistent pattern (3.5 ppm at saturation offset) for egg white protein solutions at concentrations of 30, 65, and 100% in PBS solution (pH 7), in agreement with the reported literature.^47,48^ Additionally, saturation levels for cooked egg and raw egg solutions at pH 7 were plotted, with the raw egg solution exhibiting the highest saturation. Figure 6b displays the Z-spectrum plot at different concentrations of pMAD5-based hydrogel. The degree of APT increases as the concentration of pMAD5-based hydrogel increases. The APT effect occurs at a 3.5 ppm saturation offset, and the relayed Nuclear Overhauser Effect (rNOE) at pMAD552 hydrogel indicates paired detection. These outcomes are in good agreement with the observations reported in the literature.^47^

**Figure 7:**
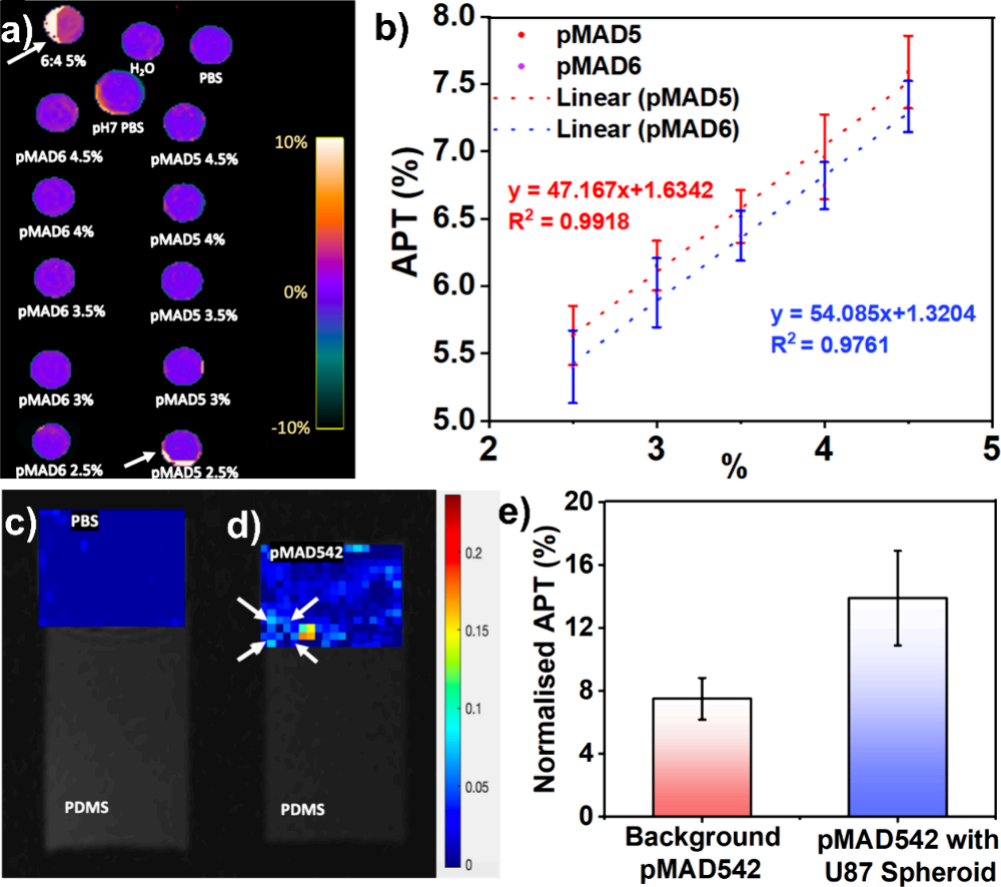
(a) APT-weighted CEST value map generated by the prototype sequence. (b) Plot showing APT-weighted CEST values measured for each hydrogel concentration along with linear fitting results for pMAD5 and pMAD6 hydrogels. APT-weighted CEST mapping of (c) PBS and (d) pMAD542 hydrogels with the U87 spheroid. (e) APT-weighted CEST measurement of the U87 spheroid phantom based on pMAD542 (*p* ≤ 0.01).

Next, APT-weighted CEST value maps of pMAD6 hydrogel at concentrations ranging from 2.5 wt % to 5 wt % with 0.5 wt % increments were measured as shown in Figure 6c, further illustrating the concentration dependency of APT-weighted CEST measurements. Figure 7a shows the APT-weighted CEST value maps generated by MRI and Figure 7b presents a plot depicting these measurements at each concentration of hydrogels, along with a linear fitting curve. As expected, the data show that a higher APT-weighted CEST value correlates with a higher concentration of pMAD hydrogels. However, the overlapping standard deviation in the measurements suggests a close relationship in the APT-weighted CEST values across each pMAD copolymer. This implies that the differences in the characteristics of pMAD copolymers could be negligible in bioimaging, and the choice of pMAD copolymer depends purely on the research purpose and application.

To further evaluate the capability of as-synthesized pMAD hydrogel for MRI, pMAD5 with the highest APT-weighted CEST effect in Z-spectrum (Figure 6b) was selected to prepare a tumor spheroid phantom. Briefly, U87 spheroids were prepared based on the literature.^59^ U87 spheroids (Figure S9) were placed in the glass vial for MRI measurement. This heightened sensitivity to proton transfer enables pMAD5 for the CEST MRI experiment, emphasizing its potential for enhanced molecular imaging. Furthermore, considering the potential clinical applications requiring injectable hydrogels, the impact of pMAD542 in this context was explored. Its ability to maintain stiffness and biocompatibility makes pMAD542 a promising candidate for injectable clinical scenarios.

The results unveiled the presence of the APT-weighted CEST effect in the pMAD542 hydrogel for MRI of U87 spheroids. Remarkably, the fact that the pMAD5-based hydrogel exhibited the highest APT-weighted CEST effect in the MRI is in agreement with the anticipated outcomes for the pMAD hydrogel. Figure 7d illustrates the APT-weighted CEST value map for the pMAD542 hydrogel as compared with PBS control with B0 image infusion (Figure 7c). The background signals for the pMAD542 hydrogel were quantified at 7%, consistent with the findings shown in Figure 7d. The normalized APT measurement is approximately 14%, indicating a 2-fold increase compared to the background pMAD5-based hydrogel signals. This highlights the potential capability of the pMAD542 hydrogel in biomedical imaging applications.

The results underscore the potential of the pMAD hydrogel as an MRI contrast agent for tumor spheroids. The nontoxic nature of the pMAD hydrogel opens avenues for extended research in cancer studies, encompassing applications such as drug delivery and the assessment of treatment responses. This contribution addresses a significant research gap by providing a novel MRI contrast solution specifically designed for small human tumor spheroids. Additionally, the APT-weighted CEST MRI technique could serve as an assessment tool to validate the research in clinical applications using pMAD hydrogels.

The MRI results demonstrate promising outcomes in imaging GBM tumor spheroids embedded in pMAD copolymer, expanding its potential clinical applications for diseases such as cancer or monitoring drug delivery progress. The potential of the pMAD copolymer as an MRI contrast agent to image cell metabolism, particularly in cancer diagnosis and treatment response, is noteworthy. However, a limitation lies in the low spatial resolution of APT-weighted CEST when imaging the pMAD copolymer with spheroids. Although quantitative results can be obtained, the presence of uncertainties may increase due to ringing artifacts noticeable in APT maps (Figure 7b), which may introduce measurement errors. Postprocessing algorithms could be applied to reduce such artifacts. This suggests that while challenges exist in achieving high spatial resolution with APT-weighted CEST, the pipeline in data analysis can contribute to mitigating uncertainties and enhancing the reliability of the pMAD copolymer for applications in MRI imaging of cell metabolism. Additionally, a comparative analysis (Tables S1 and S2) has been included in the Supporting Information to highlight the exceptional capabilities of our zwitterionic hydrogel for CEST MRI as well as to demonstrate the potential of the amino-yne click reaction discussed in this study. The future work will be focused on the in vivo study to further validate the benefits of these properties in animal MRI.

## Conclusions

In summary, a novel type of in situ-forming pMAD hydrogel was developed through a spontaneous amino-yne click reaction system under physiological conditions without the need for any catalyst. DA-PEG cross-linking, pMAD copolymer concentration, and the presence of functional groups on the pMAD copolymer play essential roles in the gelation time, mechanical properties, and swelling behavior of the pMAD hydrogel. Furthermore, the pMAD hydrogel can be formulated into an injectable system as its mechanical properties can be adjusted by varying the number of amines and zwitterionic groups in the copolymer structure and altering the amine/alkyne ratio. The pH-responsiveness of the pMAD hydrogel, particularly its hydrolysis to aldehydes and an amine group in acidic conditions (pH ≤ 5), adds another dimension to its versatility. Moreover, the zwitterionic groups of the MPC monomer, amine groups on the AE monomer, and enamine cross-linking contribute to the promising self-healing capacity of the hydrogel at room temperature. The pMAD hydrogel exhibits excellent biocompatibility properties, as demonstrated with NH-3T3 cells. The pMAD hydrogel was successfully employed to encapsulate NIH-3T3 fibroblasts, which remained viable inside the hydrogel structure for up to 7 days. Additionally, the pMAD hydrogel shows promise as a cellular scaffold for CEST MRI, broadening its potential to monitor therapeutic efficacy after implantation. We anticipate that the pMAD hydrogel based on an eco-friendly spontaneous amino-yne click reaction, with its good biocompatibility, degradability, and self-healing performance, will find significant practical applications.

## Supporting Information Available

Comparative analysis of hydrogels with the present work; comparative analysis of different click reactions with the present work; synthesis of DA-PEG cross-linker, synthesis of the pMA copolymer using radical polymerization, and mechanism of formation and degradation of enamine cross-linking; ATR-FTIR spectra of DA-PEG and PEG; ATR-FTIR spectra for copolymer pMA5; ^1^H NMR spectra for pMA5, pMA6, and PMA7 copolymers; gelation time based on the inverted vial method and the effect of DA-PEG concentration and pMA copolymer pMA5, pMA6, and pMA7; compressive mechanical properties of the pMAD hydrogel; pMAD hydrogel with 2 wt %, 1 wt %, and 0.5 wt % cross-linker concentration and 5 wt % pMA5 copolymer and injectable behavior of the pMAD550 hydrogel; mechanism of formation and degradation of enamine cross-linking; fluorescence images of NIH-3T3 were encapsulated inside the hydrogel in 3, 5, and 7 days by using the LIVE/DEAD cell viability kit; fluorescence images of NIH-3T3 were encapsulated inside the hydrogel in DMEM (10 % PBS) after 1, 3, and 5 days by using the LIVE/DEAD cell viability kit; and representative image of the U87 tumor spheroid (PDF)
