# Supplementary material for: Formation of Zwitterionic and Self-Healable Hydrogels via Amino-yne Click Chemistry for Development of Cellular Scaffold and Tumor Spheroid Phantom for MRI
Source: ACS Appl Mater Interfaces. 2024 Jul 8;16(28):36157–67. doi: 10.1021/acsami.4c06917 (PMC11261563; doi:10.1021/acsami.4c06917)
Supplement: Supplementary file 1 — am4c06917_si_001.pdf [file am4c06917_si_001.pdf]

Supporting Information

Formation of Zwitterionic and Self-healable Hydrogels via Amino-yne Click Chemistry for Development of Cellular Scaffold and Tumor Spheroid Phantom for MRI

Cao Tuong Vi Nguyen [a§], *Steven Kwok Keung Chow* [c§], *Hoang Nam Nguyen* [a§], *Tesi Liu* [d], *Angela Walls* [c], *Stephanie Withey* [e], *Patrick Liebig* [f], *Marco Mueller* [g], *Benjamin Thierry* [d], *Chih-Tsung Yang* [d]*, *Chun-Jen Huang* [a, b]*

[a] Department of Chemical & Materials Engineering, National Central University, Jhong-Li, Taoyuan 320, Taiwan.

[b] R&D Center for Membrane Technology, Chung Yuan Christian University, 200 Chung Pei Rd., Chung-Li City 32023, Taiwan.

[c] Clinical Research and Imaging Centre, South Australian Health and Medical Research Institute, Australia.

[d] Future Industries Institute, University of South Australia, Mawson Lakes Campus, Adelaide, SA 5095, Australia

[e] Siemens Healthcare Pty Ltd, Adelaide, Australia

[f] Siemens Healthcare GmbH, Erlangen, Germany

[g] Advanced Clinical Imaging Technology, Siemens Healthineers International AG, Lausanne, Switzerland

§ These authors contributed equally to this work.

* Corresponding author. E-mail: chih-tsung.yang@unisa.edu.au (CJY), cjhuang@ncu.edu.tw (CJH)

Table S1: Comparative Analysis of Hydrogels with Present work.

Hydrogel	pH responsive	Self-healable	Clinical MRI (3T)	Biocompatible/ non-fouling	Drug carrier	Ref
Zwitterionic	✓	✓	✓	✓	✓	This study
Chitosan-dextran	x	✓	✓	✓	✓	1
Alginate microbeads	✓	x	✓	✓	✓	2
Peptide	x	✓	x	✓	✓	3
Liposome	x	✓	✓	✓	✓	4

Table S2: Comparative Analysis of Different Click Reactions with Present Work.

Reaction	Solvent	Stiffness	Catalyst	Self-healing	Degradati on	Ref
Amino-yne	PBS	✓	x	✓	✓	This study
Thiol-yne	PBS	x	x	x	x	5
Azide-Alkyne Cycloaddition	Water/EtOH	✓	CuSO4 sodium ascorbate	x	x	6
Thiol-ene	PBS	x	UV-irradiation	✓	✓	7
Diels–Alder	HCl (pH = 5)	✓	65^0C	✓	x	8

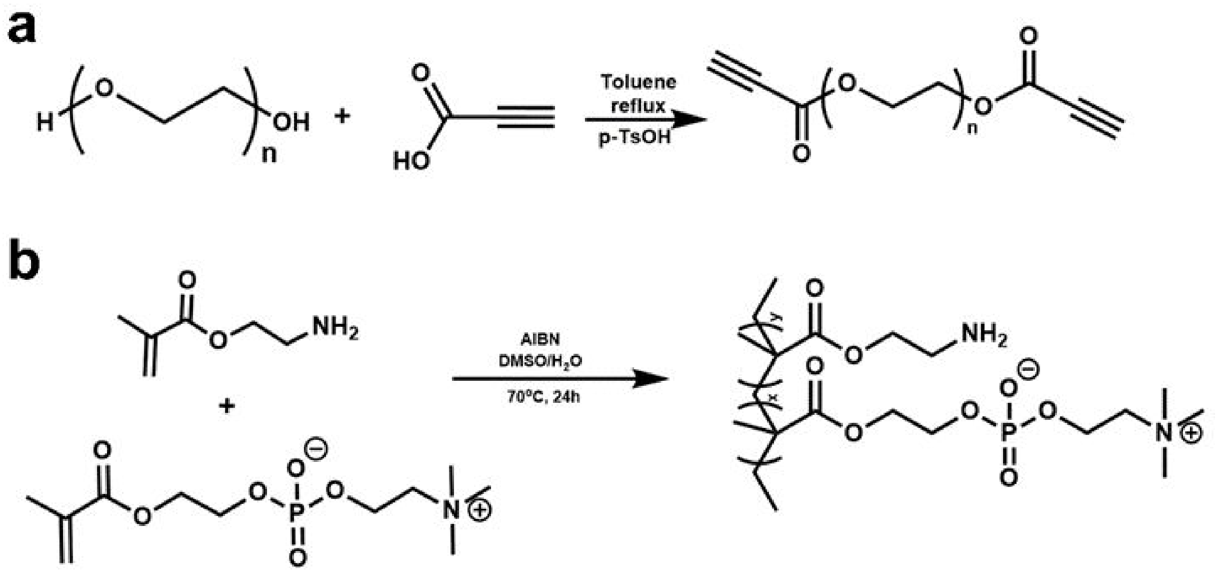

Scheme S1: a) synthesis DA-PEG crosslinker, b) synthesis pMA copolymer using radical polymerization, c) mechanism of formation and degradation of enamine crosslinking.

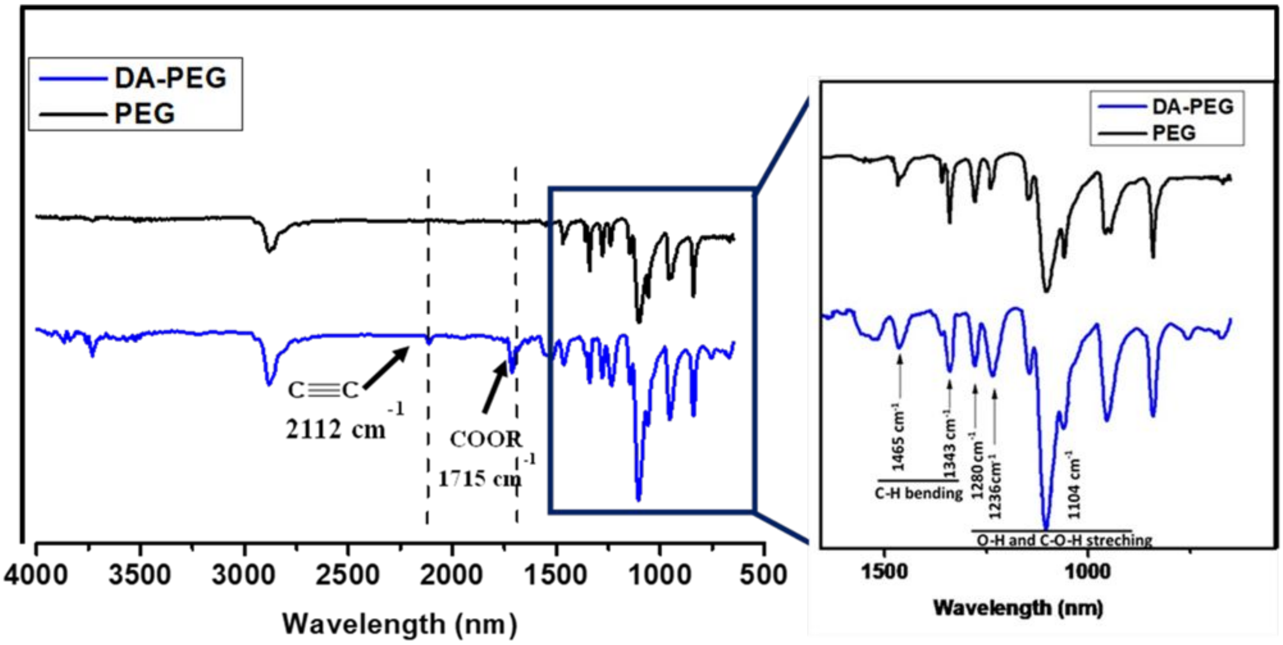

Figure S1. ATR-FTIR spectra of DA-PEG and PEG

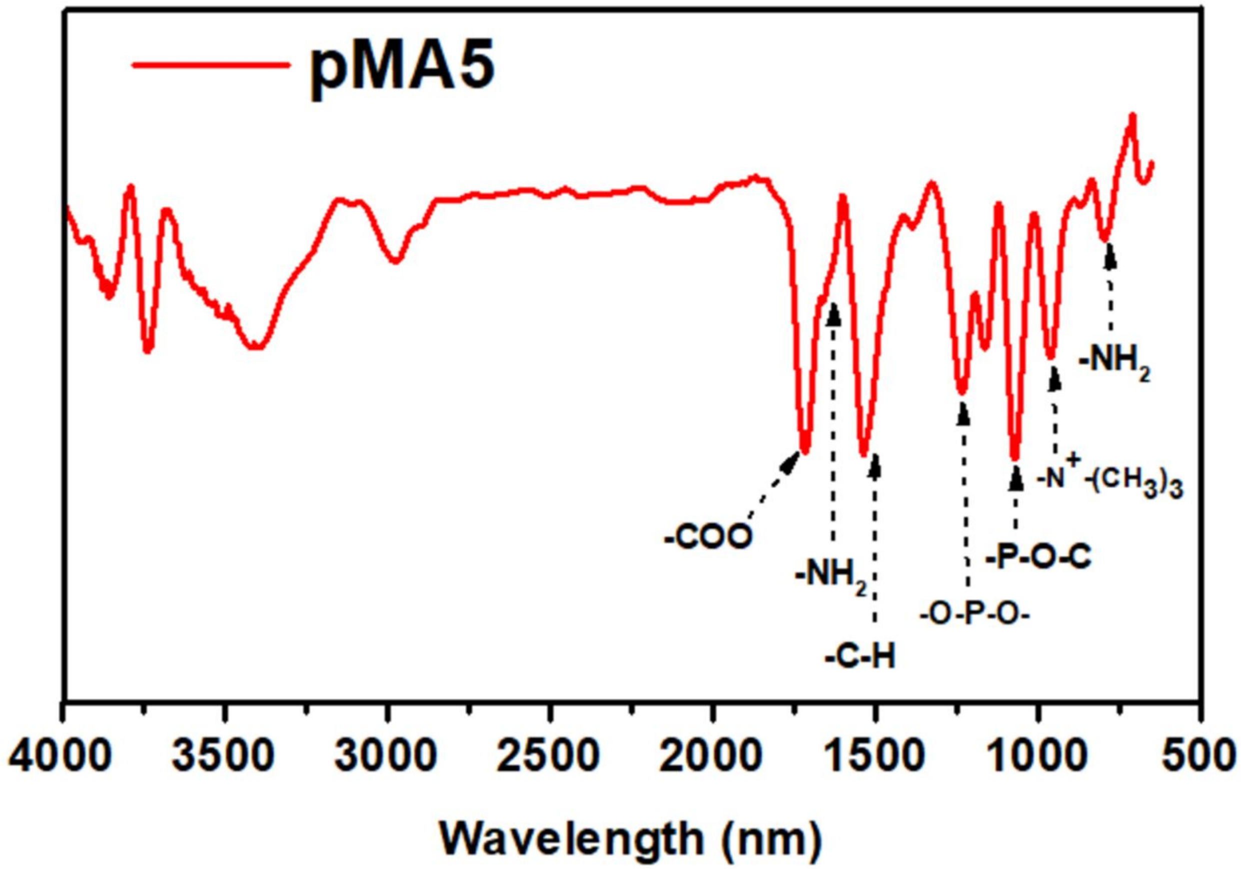

Figure S2: ATR-FTIR spectra for copolymer pMA5

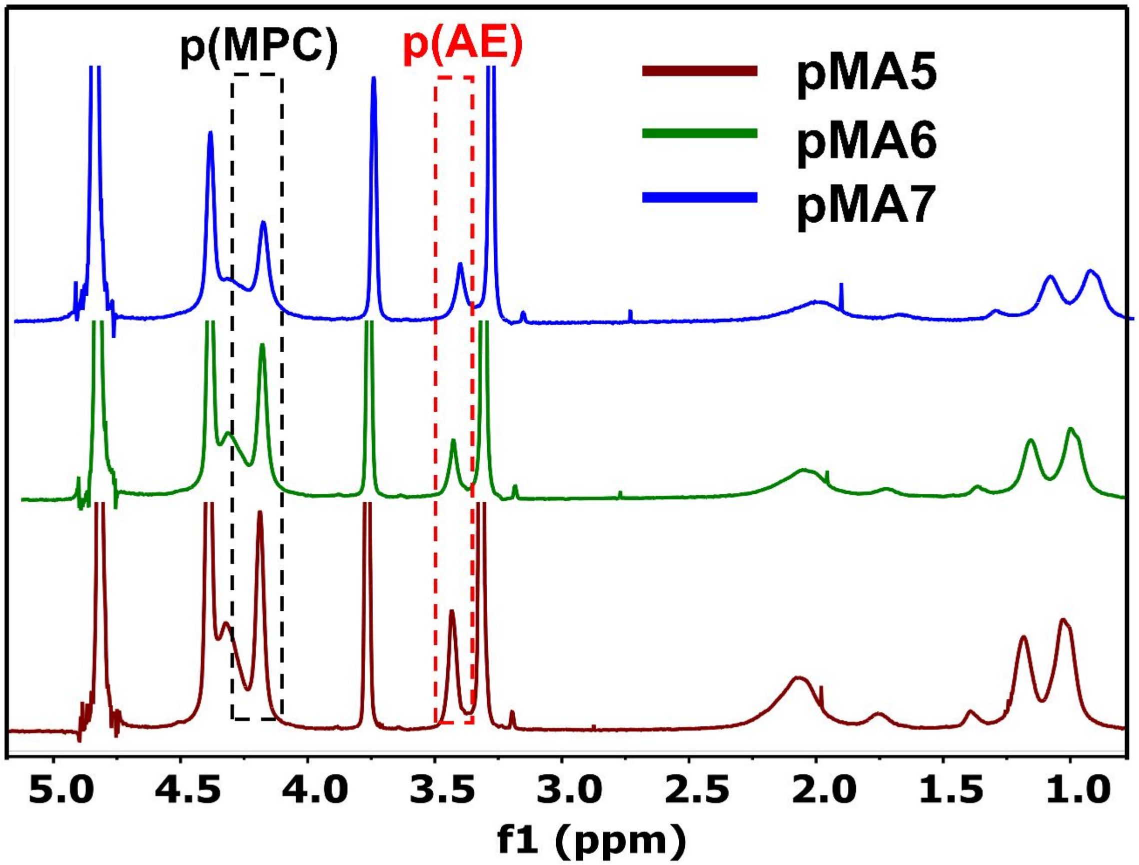

Figure S3. ^{1}H NMR spectrum for pMA5, pMA6 and PMA7 copolymer.

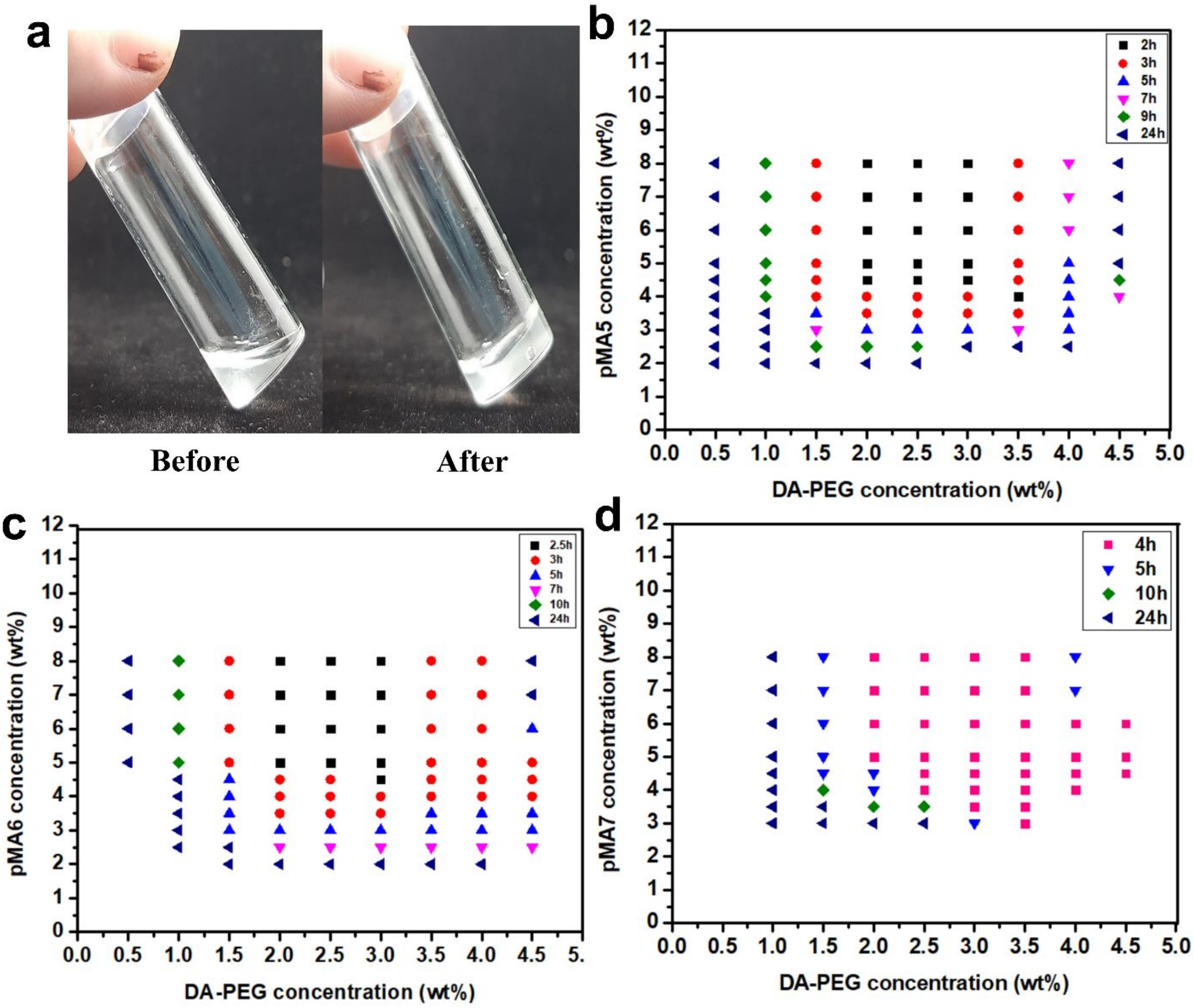

Figure S4 a) Gelation time based on inverted vial method. Effect of DA-PEG concentration and pMA copolymer b) pMA5, c) pMA6, d) pMA7.

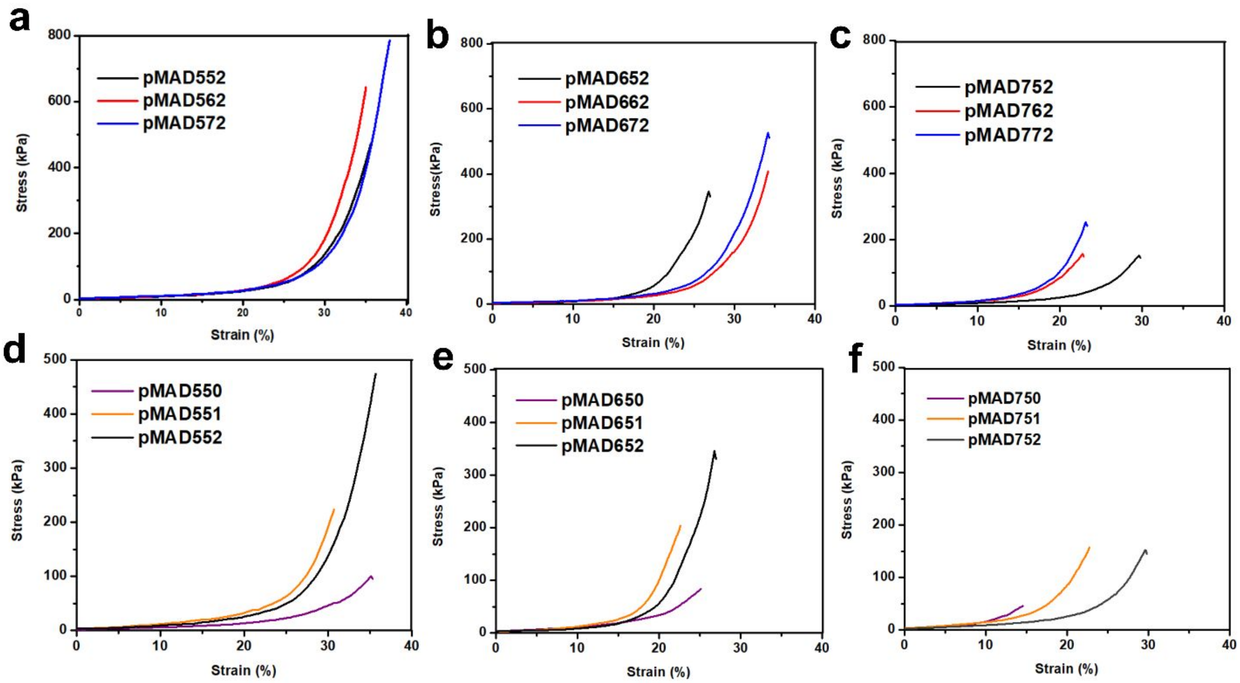

Figure S5. Compressive mechanical properties of pMAD hydrogel

a-c) Comparison of compressive stress and strain pMAD with different pMA copolymer concentration including 5wt%, 6 wt% and 7 wt%, respectively.

d-f) Compare compressive stress between 5 wt% of pMA copolymer and three concentration of DA-PEG crosslinker which are 0.5 wt%, 1 wt% and 2 wt%, respectively.

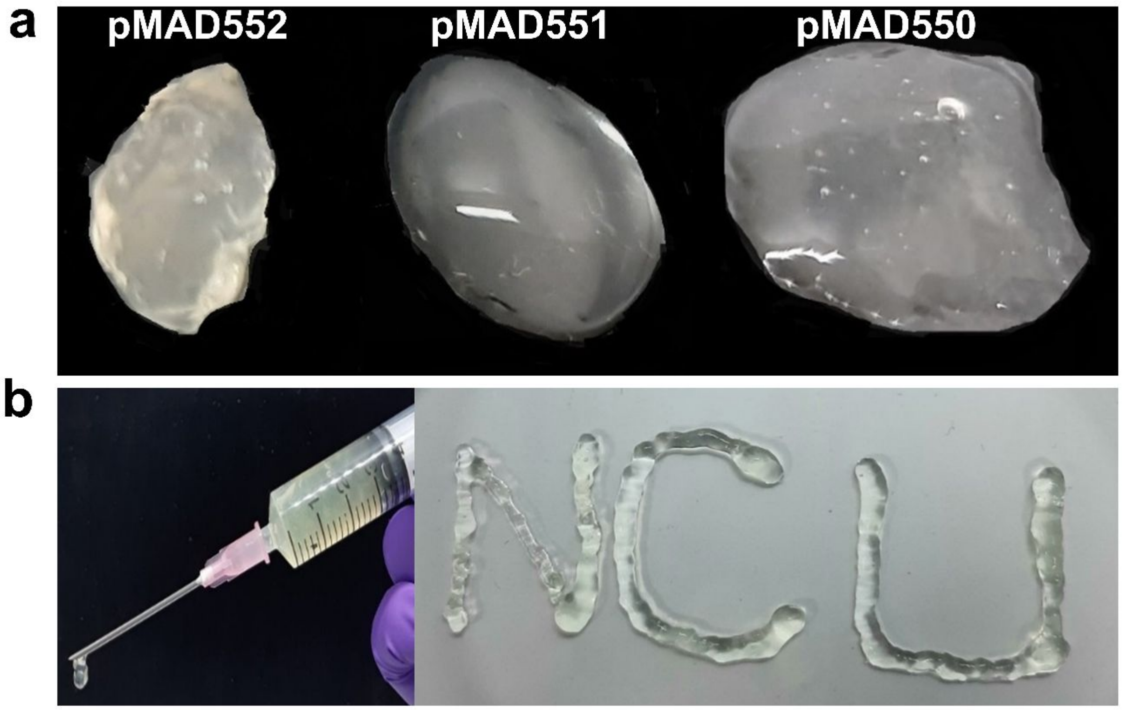

Figure S6. pMAD hydrogel with 2wt%, 1wt% and 0.5 wt% crosslinker concentration and 5wt% pMA5 copolymer, b) injectable behavior of pMAD550 hydrogel

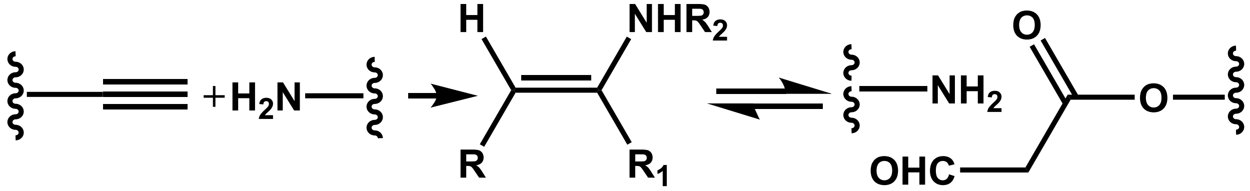

Scheme S2. Mechanism of formation and degradation of enamine crosslinking.

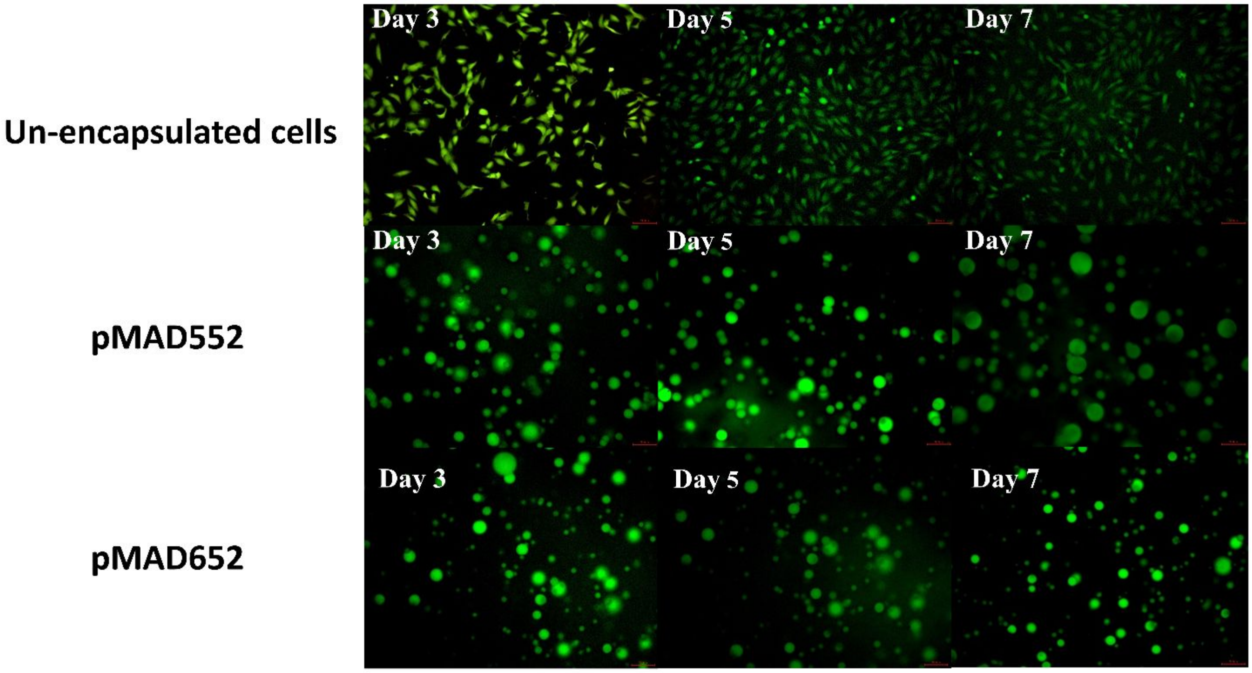

Figure S7. Fluorescence images of NIH-3T3 were encapsulated inside hydrogel in 3,5 and 7 days by using LIVE/DEAD cell viability Kit. (Scale bar: 100μm)

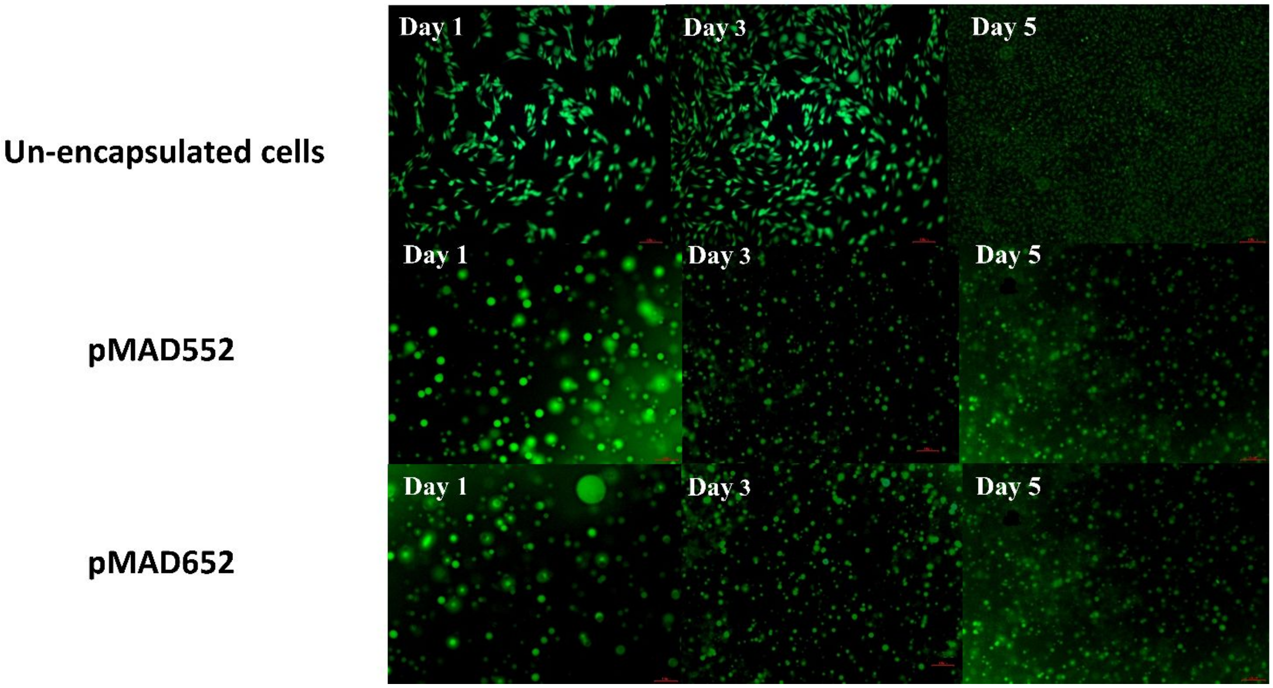

Figure S8. Fluorescence images of NIH-3T3 were encapsulated inside hydrogel in DMEM (10 % PBS) after 1,3 and 5 days by using LIVE/DEAD cell viability Kit. (Scale bar: 100 μm)

Preparation of U87 tumor spheroids

U87 cells were trypsinized and resuspended in complete DMEM medium. 25,000 cells in 25 μL of medium was dispensed as one droplet on the lid of 100 mm cell culture dish, while 2 mL of the PBS was added in the bottom of the dish to maintain the moisture. Small spheroids were formed after 24 h of culture, which were subsequently transferred to a 6-well plate for further growth. 2 mL of complete DMEM medium was added in each well and the plate was placed on a shaker set at 80 rpm in incubator to culture for 7 days until the spheroids reached approximate 1.5 mm in diameter.

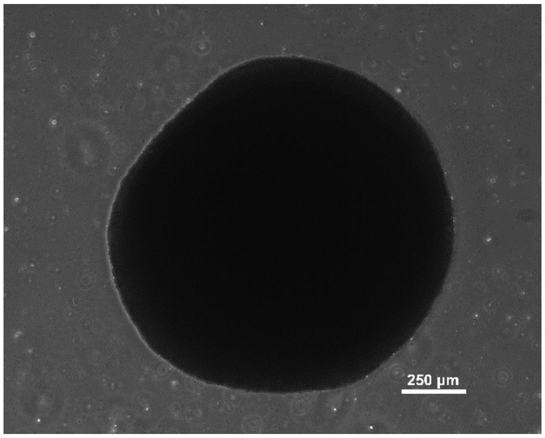

Figure S9. Representative image of U87 tumor spheroid.

;

REFERENCES

(1) Han, X.; Lai, J. H. C.; Huang, J.; Park, S. W.; Liu, Y.; Chan, K. W. Y. Imaging Self-Healing Hydrogels and Chemotherapeutics Using CEST MRI at 3 T. *ACS Appl Bio Mater* **2021**, *4* (7), 5605-5616. DOI: 10.1021/acsabm.1c00411.
(2) Xiao, P.; Huang, J.; Han, X.; Cheu, J. W. S.; Liu, Y.; Law, L. H.; Lai, J. H. C.; Li, J.; Park, S. W.; Wong, C. C. L.; et al. Monitor Tumor pHe and Response Longitudinally during Treatment Using CEST MRI-Detectable Alginate Microbeads. *ACS Appl. Mater. Interfaces.* **2022**, *14* (49), 54401-54410. DOI: 10.1021/acsami.2c10493.
(3) Rosa, E.; Di Gregorio, E.; Ferrauto, G.; Diaferia, C.; Gallo, E.; Terreno, E.; Accardo, A. Hybrid PNA-peptide hydrogels as injectable CEST-MRI agents. *J Mater Chem B* **2024**. DOI: 10.1039/d4tb00358f.
(4) Park, S. W.; Lai, J. H. C.; Han, X.; Leung, V. W. M.; Xiao, P.; Huang, J.; Chan, K. W. Y. Preclinical Application of CEST MRI to Detect Early and Regional Tumor Response to Local Brain Tumor Treatment. *Pharmaceutics* **2024**, *16* (1). DOI: 10.3390/pharmaceutics16010101.
(5) Macdougall, L. J.; Truong, V. X.; Dove, A. P. Efficient In Situ Nucleophilic Thiol-yne Click Chemistry for the Synthesis of Strong Hydrogel Materials with Tunable Properties. *ACS Macro Lett* **2017**, *6* (2), 93-97. DOI: 10.1021/acsmacrolett.6b00857.
(6) Piluso, S.; Vukicevie, R.; Nöchel, U.; Braune, S.; Lendlein, A.; Neffe, A. T. Sequential alkyne-azide cycloadditions for functionalized gelatin hydrogel formation. *Eur Polym J* **2018**, *100*, 77-85. DOI: 10.1016/j.eurpolymj.2018.01.017.
(7) Sawicki, L. A.; Kloxin, A. M. Design of thiol-ene photoclick hydrogels using facile techniques for cell culture applicationsdaggerElectronic supplementary information (ESI) available. See DOI: 10.1039/c4bm00187gClick here for additional data file. *Biomater Sci* **2014**, *2* (11), 1612-1626. DOI: 10.1039/c4bm00187g.
(8) Li, D. Q.; Wang, S. Y.; Meng, Y. J.; Guo, Z. W.; Cheng, M. M.; Li, J. Fabrication of self-healing pectin/chitosan hybrid hydrogel via Diels-Alder reactions for drug delivery with high swelling property, pH-responsiveness, and cytocompatibility. *Carbohydr Polym* **2021**, *268*, 118244. DOI: 10.1016/j.carbpol.2021.118244.
